# Surgical and functional impact of nerve-sparing radical hysterectomy for parametrial deep endometriosis: a single centre experience

**DOI:** 10.52054/FVVO.14.2.016

**Published:** 2022-07-01

**Authors:** A Rosati, M Pavone, F Campolo, A De Cicco Nardone, D Raimondo, R Seracchioli, G Scambia, M.M. Ianieri

**Affiliations:** Division of Gynecological Oncology, Department of Women’s and Children’s Health, Fondazione Policlinico Universitario A. Gemelli IRCCS, Rome, Italy; Division of Gynaecology and Human Reproduction Physiopathology, IRCCS University Hospital of Bologna, Bologna, Italy; Department of Medical and Surgical Sciences, University of Bologna, Bologna, Italy

**Keywords:** radical hysterectomy, deep infiltrative endometriosis, parametrectomy, nerve-sparing, functional outcome

## Abstract

**Background:**

Deep endometriosis (DE) usually creates a distortion of the retroperitoneal anatomy and may infiltrate the parametria with an oncomimetic pathway similar to cervical cancer. The condition represents a severe manifestation of endometriosis that may result in a functional impairment of the inferior hypogastric plexus. An extensive surgical resection may be required with an associated risk of increased neurogenic postoperative pelvic organ dysfunction.

**Objectives:**

To evaluate the post-operative function and complications following hysterectomy with posterolateral parametrial resection for DE.

**Materials and Methods:**

In total, 23 patients underwent radical hysterectomy for DE with the parametria involved. The severity of pain was assessed by the Visual Analogue Scale (VAS) score. The KESS, GQLI, BFLUTS and FSFI were used to examine the gastrointestinal, urinary and sexual functions respectively. Intra and post-operative complications were recorded.

**Main outcome measures:**

The main outcomes were gastrointestinal, urinary and sexual function and intra and post-operative complications.

**Results:**

Dyschezia, dyspareunia and chronic pelvic pain were significantly reduced following hysterectomy. Furthermore, an improvement of gastrointestinal function was observed, while sexual functions, examined by FSFI and urinary symptoms, examined by BFLUTS, was not shown to be significant.

**Conclusion:**

The modified nerve-sparing radical hysterectomy for DE results in an improvement of symptoms. Nevertheless, despite the nerve-sparing approach, this procedure may be associated with a not-negligible risk of post-operative bladder voiding deficit.

**What is new?:**

This is the first study that focuses on parametrial endometriosis using validated questionnaires to assess functional outcomes following radical hysterectomy for DE.

## Introduction

Endometriosis is a common non-malignant gynaecologic disease, defined as the presence of ectopic endometrial glands and stroma. Essentially, three types of lesions are reported: ovarian, peritoneal, and deep endometriosis (DE) (Ianieri et al., 2018).

DE usually creates a distortion of the retroperitoneal anatomy similar to that occurring in cervical cancer, and this infiltrative pathway may therefore be defined as “oncomimetic” (Mabrouk et al., 2019).

Parametrial infiltration, and specifically the lateral parametrium invasion, occurs in approximately 17% of DE cases (Mabrouk et al., 2019). This condition correlates with the most severe manifestations of the disease such as ureteral stenosis, resulting in bladder emptying disorders and constipation, mainly due to the functional impairment of the inferior hypogastric plexus (Chiantera et al., 2018). The parametrial DE may require an extensive retroperitoneal dissection, however, this approach is known to be associated with a considerable risk of neurogenic postoperative pelvic organ dysfunction (Darai et al., 2005).

The anatomy of parametria has not yet been clearly defined in the literature (Chiantera et al., 2018; Ercoli et al., 2005; Querleu et al., 2017). As previously reported (Ercoli et al., 2005; Ceccaroni et al., 2013), the lateral and posterior parametria consist of an area of connective tissue surrounding the lympho-vascular trunks that supports pelvic viscera. This bundle of variably dense connective tissue is part of the extra serosal pelvic fascia (Ercoli et al., 2005).

In selected cases of DE, parametrial resection using nerve-sparing techniques is required during hysterectomy; these have been adopted from oncologic procedures as seen in cervical cancer (Raspagliesi et al., 2017). These principles have been applied over the years to reduce the incidence of post-operative functional impairment of pelvic organs (Kyo et al., 2016).

To date, the literature lacks studies describing the anatomical landmarks, the surgical steps and the overall functional complications of the nerve- sparing radical hysterectomy (NSRH) for DE.

Hence, the aim of our study was to evaluate the post-operative functional damage and complications of hysterectomy with posterolateral parametrial resection for DE.

## Materials and Methods

### 1. Patient selection

For this retrospective observational study approval by the Institutional Review Board was obtained (number: DIPUSVSP-27-07-20104) and written informed consent was signed by all patients.

The study was conducted at the Fondazione Policlinico Universitario A. Gemelli IRCCS, which is a third level centre for the treatment of endometriosis.

All data from patients who underwent minimally invasive nerve-sparing radical hysterectomy (NSRH) for DE, between May 2019 and December 2020 were collected. Patients were included if previous medical therapy failed.

Inclusion criteria were: age ≥ 18 years, patients with suspected parametrial DE at pre-operative evaluation, patients subjected to minimally invasive nerve-sparing radical hysterectomy, diagnosis of endometriosis at final pathological evaluation.

Exclusion criteria for the present study were: procedures other than NSRH such as bowel, bladder or ureteral resections, diagnosis of multiple sclerosis, pre- or post-operative absence of sexual activity, gross involvement of hypogastric nerves or inferior hypogastric plexus during surgical dissection; pre-operative urodynamic diagnosis of neurogenic bladder or bladder dysfunction, the laparotomic approach and refusal to answer the proposed questionnaires.

During preoperative evaluation, medical and surgical history were recorded. All women were subjected to recto-vaginal examination, advanced transvaginal ultrasonography and/or pelvic magnetic resonance imaging.

Data on medical history (age, body mass index, previous surgery), the clinical variables (pain symptoms, urinary, gastrointestinal and sexual function), the surgical findings (operating time, estimated blood loss, any intraoperative complications), and the peri-operative variables (days of hospitalisation, need for self- catheterisation, postoperative complications) were recorded in a database.

Early postoperative complications, defined as complications which occurred within 30 days after surgery, were registered and described according to the Extended Clavien-Dindo classification of surgical complications (Dindo et al., 2004).

### 2. Outcome assessment

The severity of pain symptoms (dysmenorrhea, dysuria, dyschezia, dyspareunia and chronic pelvic pain) were assessed by the Visual Analogue Scale (VAS) score (ranging from 0 to 10, no pain to most severe pain respectively).

Information about functional outcomes were assessed using validated questionnaires: the Knowles-Eccersley-Scott-Symptom Questionnaire (KESS), the Gastrointestinal Quality of Life Index (GQLI) (Nieveen Van Dijkum et al., 2000), the Bristol Female Lower Urinary Tract Symptoms questionnaire (BFLUTS) (Brookes et al., 2005) and the Female Sexual Function Index (FSFI) (Filocamo et al., 2014).

The KESS questionnaire was used to assess the bowel function and specifically to determine whether the patient suffered from constipation (0 to 39 points). We used a cut-off criterion of >-10 points in the total KESS score to define constipation (Knowles et al., 2000).

The GQLI was used to describe health- related quality of life (QoL) of patients with gastrointestinal disease (0 to 144 points). This questionnaire consists of 36 questions with a total score varying from 0 to 144, in which a higher score indicates a better QoL (Nieveen Van Dijkum et al., 2000).

The BFLUTS was used to assess urinary function (Knowles et al., 2000), with a total score ranging from 0 to 45, where higher scores imply a decreased bladder function.

Finally, for the assessment of sexual function we applied the validated Italian translation of the FSFI. It is composed of 19 questions exploring all domains of sexual function. A total FSFI score of less than 26.5 was considered as female sexual dysfunction (Filocamo et al., 2014).

Both the questionnaires and the assessment of pain scores were carried out at pre-operative evaluation and 6 months following surgery.

Urinary retention was defined by a post-voiding residual volume of 100 ml. In those cases, self- catheterisation was recommended until the post urinary residual volume was <100 ml for three consecutive measurements.

### 3. Surgical technique

All patients were operated at “Fondazione Policlinico Universitario A. Gemelli IRCCS, Rome” by the same surgeon (MMI) whom has widely recognised experience in radical pelvic surgery.

Each procedure was performed using a minimally-invasive approach.

#### Nerve-sparing radical hysterectomy for DE

All surgeries were performed using the same standard steps. The abdomen was accessed by abdominal insufflation with a Veress needle or with an optical trocar. Three 5-mm accessory ports were subsequently placed in the left lower quadrant, right lower quadrant and supra-pubic area under direct visualisation. A uterine manipulator was used in every case.

**Figure 1:**
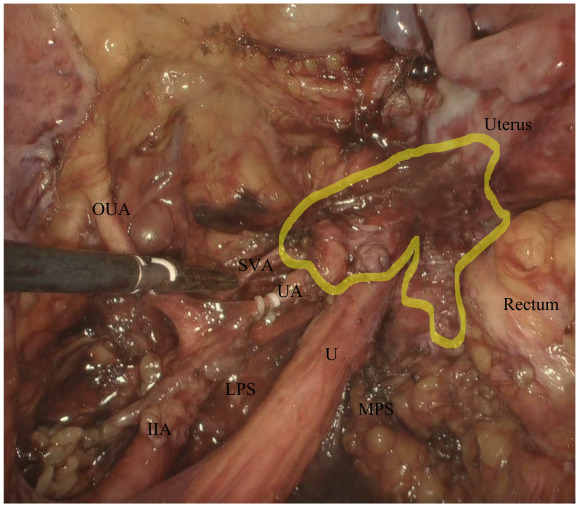
Closing of the uterine artery at its origin and development of medial and lateral pararectal spaces. The highlighted area identifies the endometriotic nodule infiltrating the posterolateral parametrium. IIA: internal iliac artery U: ureter OUA: obliterated umbelical artery UA: uterine artery SVA: superior vesical artery MPS: medial pararectal space LPS: lateral pararectal space

**Figure 2:**
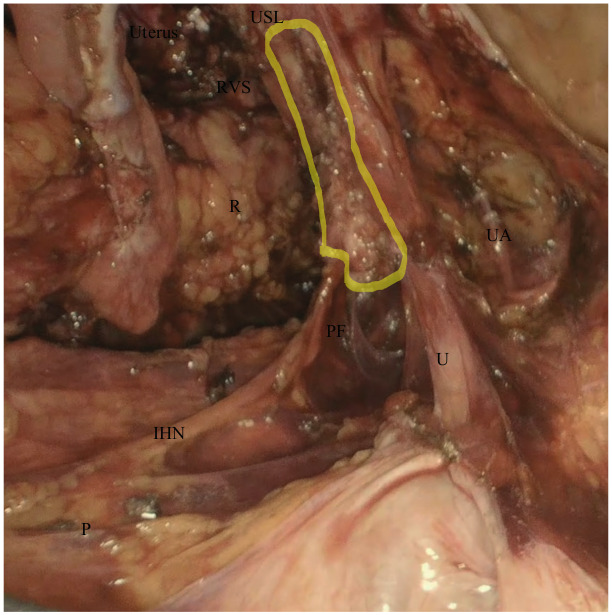
Individuation of the inferior hypogastric nerve preserving presacral fascia and isolation of an endometriotic nodule infiltrating the posterior parametrium. The highlighted area identifies the endometriotic nodule infiltrating the posterior parametrium. P: promontorium IHN: inferior hypogastric nerve U: ureter PF: presacral fascia R: rectum UA: uterine artery USL: utero-sacral ligament RVS: recto-vaginal space

**Figure 3:**
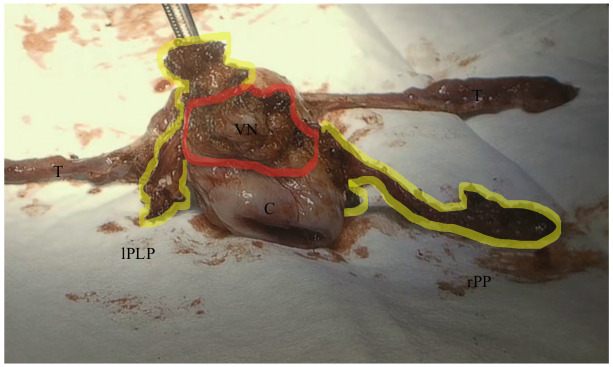
Specimen of nerve-sparing radical hysterectomy: en bloc parametrectomy and vaginal nodule resection. The yellow highlighted area on the right identifies endometriotic nodules infiltrating the right posterior parametrium (rPP) and on the left, the left postero-lateral parametrium (lPLP). The red highlighted area identifies the vaginal and recto-vaginal space endometriotic nodule. VN: vaginal nodule C: cervix T: tubes

The procedure began with adhesiolysis and ovarian surgery (drainage and stripping of endometriomas) when required. Afterwards, access to the retroperitoneum was obtained. For each patient we followed the same steps, namely:

- Coagulation and transection of both round ligaments. Incision of the anterior fold of broad ligament and identification of the obliterated umbilical artery, uterine artery and deep uterine vein (Figure 1).Coagulation and transection of both round ligaments. Incision of the anterior fold of broad ligament and identification of the obliterated umbilical artery, uterine artery and deep uterine vein (Figure 1).
- Development of medial pararectal spaces (Okabayashi’s space), between the presacral fascia covering the ureter (lateral) and the uterosacral ligament (medial) and the lateral pararectal space (Latzko’s space), between the presacral fascia covering the ureter (medial) and the internal iliac vessels (lateral) (Figure 1).
- Development of the medial paravesical space, when needed, between the umbilical artery (lateral) and the lateral wall of bladder (medial).
- Incision of the vesico-uterine fold and , after filling the bladder with 180 ml of saline solution, developing the vesico-uterine space until 1-2 cm of vagina was exposed.
- Skeletonisation, closing and cutting of the uterine artery at its origin.
- Lateralisation of both ureters or ureterolysis in case a DE nodule was present that involved the ureter, using a medial approach.
- Lateralisation of the hypogastric nerves which were preserved if possible, the presacral fascia and development of recto-vaginal space in a latero-medial approach (Figure 2).
- The nerve-sparing parametrectomy included the dissection of the posterior (utero-sacral ligament, lateral ligaments of the rectum, recto- vaginal ligaments) and lateral parametrium (surrounding the uterine vessels and the ureter, up to the hypogastric fascia covering the splanchnic nerves) to a variable extent. These procedures were performed to spare the ortho- and parasympathetic fibers of the inferior hypogastric plexus. Pelvic splanchnic nerves were identified in cases where the DE nodule infiltrated the presacral fascia wherein the inferior hypogastric nerve is located.
- Colpotomy and uterus removal with en bloc parametria and vaginal nodule (when present) (Figure 3). The vagina was closed laparoscopically with two hemi-continuous running polyfilament sutures.

### 4. Statistical analysis

Statistical analyses were performed using SPSS version 27.0 (IBM, Armonk, New York, USA). Clinical and demographic characteristics were described as descriptive statistics. Qualitative variables were described with percentages and quantitative variables with mean ± standard deviation.

Comparisons between categorical variables were performed with χ2 test or Fisher Exact Test. Continuous variables were compared using either the Student T test when data were normally distributed, or the Mann-Whitney test when data were not normally distributed. The Wilcoxon signed-rank test was used to compare continuous variables among the same group. P-values <0.05 were considered significant for all tests.

Since this investigation was designed as an exploratory study, a sample size calculation was not performed.

## Results

All patients undergoing NSRH for DE who met the inclusion criteria were included in our study. Among the 23 patients the median age was 43 years old (38-46) and median body mass index (BMI) was 23.4 (21.3-25) kg/m2.

A significant number of patients (16) had undergone previous abdominal surgery, of whom eight (34%) had previous retroperitoneal surgery for DE without bowel or bladder involvement, three (13%) had laparoscopic adhesiolysis and enucleation of an endometrioma, and five (21%) had abdominal surgery not related to endometriosis.

With respect to the disease stage, the majority of patients (70%) had stage 3 disease and 7 out of 23 patients (30%) had stage 4 disease, according to the r-ASRM classification (Revised American Society for Reproductive Medicine classification of endometriosis, 1997).

The surgical route was always minimally invasive and no conversion to laparotomy was needed.

All women had a histological diagnosis of endometriosis and underwent either unilateral (30.4%) or bilateral (69.4 %) posterolateral parametrectomy.

Clinical and demographic characteristics, as well as the description of operative surgical procedures are listed in Table I. Type B radical hysterectomy was executed in 70% of patients and type C1 radical hysterectomy in 30% of patients, according to the Querleu-Morrow classification (Querleu et al., 2017).

**Table I:** Baseline characteristics and surgical procedures.

Age (median, range)	43 (38-46)
BMI (median, range)	23.4 (21.3-25)
Previous pelvic surgery (n,%)	Previous pelvic surgery (n,%)
Type of parametrectomy (n,%)	
Type A	-
Type B	16 (69.6)
Type C	17 (30.4)
Unilateral parametrial resection	7 (30.4)
BSO	6 (26.1)
Excision of ovarian endometrioma	17 (73.9)

Body Mass Index (BMI), bilateral salpingo-oophorectomy (BSO)

Bilateral salpingo-oophorectomy was performed in 26% of patients. An ovarian endometrioma was excised in 74% of patients and bilateral salpingectomy was performed in 65% of patients.

All perioperative variables and postoperative complications are listed in Table II.

**Table II:** Baseline characteristics and surgical procedures.

EBL (median, range*)	150 (50-200)
Operative time (median, range*)	219 (180-316)
Days of hospitalisation (median, range*)	5 (3-6)
Fever (n,%)	1 (4.3)
Urinary tract infection (n,%)	3 (13)
Bladder voiding deficit (n,%)	3 (13)
Clavien-dindo (n,%)	
G1	1 (4.3)
G2	7 (30.4 )
G3	0

*Range expressed as Q1–Q3; Estimated blood loss (EBL)

The median blood loss was 150 ml (range 50-200) and median operative time was 219 minutes (range 180- 316).

Postoperative complications included fever, reported in one patient (4%), bladder voiding deficit and urinary tract infections, both reported in three patients (13%). Among the women who experienced post-voiding deficit, only one required self-catheterisation for three months. All patients recovered spontaneously, in less than 30 post-operative days.

No “severe” postoperative complications, defined as Clavien-Dindo grade more than III were reported.

Table III shows functional outcomes and VAS scores before and after surgery.

**Table III:** Functional outcomes and VAS scores before and after surgery.

	Preoperative (Mean, SD)	Postoperative (Mean, SD)	p value
Dyschezia VAS	2.91 + - 0.688	0.65 + - 0.36	**0.02**
Dysuria VAS	1.13 + - 0.53	0.7 + - 0.48	0.611
Dyspareunia VAS	3.65 + - 0.76	1.57 + - 0.54	**0.012**
Chronic pelvic pain	2.91 + - 0.76	0.78 + - 0.37	**0.012**
KESS	14.35 + - 1.6	8.48 + - 1.07	**0.002**
GQLI	88.3 + - 3.3	105.17 + - 3.23	**0.0001**
FSFI	21.4 + - 2.32	20.47 + - 2.4	0.98
BFLUTS	5.35 + - 1.32	5.26 + - 0.71	0.46

There was a statistically significant decrease from pre- to post-intervention in mean dyschezia (pre: 2.91 + - 0.688 post: 0.65 + - 0.36, p=0.02), dyspareunia (pre: 3.65 + - 0.76 post: 1.57 + - 0.54, p= 0.012), chronic pelvic pain (pre: 2.91 + - 0.76 post: 0.78 + - 0.37, p=0.012) and KESS (pre:14.35 + - 1.6 post:8.48 + - 1.07, p=0.002). Moreover, there was a significant increase in mean GQLI (pre: 88.3 + - 3.3 post: 105.17 + - 3.23, p=0.0001), representing an improvement of gastrointestinal functions. FSFI and BFLUTS, representing sexual and urinary functions respectively did not change over time nor was there a significant change seen in female sexual dysfunction (pre: 52.2% post: 69.6%, p = 0.344).

The constipation rate was highly reported in the study population both at baseline evaluation (70% patients) and after surgery (58% of patients). Nonetheless, these rates were not significantly different (p = 0.07).

## Discussion

In our study, surgical eradication of parametrial DE led to a general improvement of pain scores and gastrointestinal function. However, despite a nerve-sparing approach for parametrial resection, post-operative bladder voiding deficit was frequently reported. This finding is in line with results reported in the literature, where the incidence of post-operative bladder dysfunction requiring self-catheterisation was one of the most frequent complications (Ballester et al., 2011; Uccella et al., 2018; Nezhat et al., 2020).

Ballester et al. (2011) reported a self- catheterisation incidence of 37% during the immediate postoperative period and the need for bladder catheterisation in 12.5% of patients over a 3-month follow-up period.

Uccella et al. (2018) analysed the functional outcome of nerve-sparing DE eradication and observed that 11.8% of patients experienced urinary retention immediately after surgery, with spontaneous improvement in all patients.

Nezhat et al. (2020) investigated the post- operative complication rate following the modified radical hysterectomy for DE and reported urinary retention in 0.9% of cases. However, this finding is difficult to interpret due to the lack of a standardised classification of parametrial resection for DE.

The parametria can be considered the “neural core” of the pelvis. The pelvic splanchnic nerves, originating from S2-S3-S4, pierce the hypogastric fascia and join in a latero-median way to the hypogastric nerves, to form the inferior hypogastric plexus (IHP).

The efferent branches of the IHP can be divided in three groups of fibers: one directs medially to the rectum passing through the lateral ligament of the rectum; another directs to the uterus running through the cardinal ligament; and the third to the bladder and vagina running caudally through the paracervix up to the so-called ‘‘ventral parametrium’’ (Ceccaroni et al., 2013). The preservation of these fibers may avoid bladder and rectal dysfunction and prevent reduction in vaginal lubrification and arousal (Ceccaroni et al., 2016).

Our analysis showed a significant improvement over time in KESS and GIQLI after surgery for DE, but not for BFLUTS and FSFI. These data contrast other authors, in which the eradication of DE did not guarantee relief from digestive complains. These differences might be due to the absence of patients with bowel endometriosis in our series (Kupelian and Cutner, 2016; Riiskjaer et al., 2018; D’Avout-Fourdinier et al., 2020).

Uccella et al. (2018) reported an improvement in dyspareunia following surgery, however there was not a significant improvement seen in terms of FSFI in our study.

This may be explained by the inevitable stretching of the nerve fibers when a radical parametrectomy is performed, even when a nerve-sparing approach is applied (Ceccaroni et al., 2016).

We share Darwish and Roman’s perplexities regarding the possibility to perform nerve-sparing surgery in cases of bilateral parametrial involvement that “are more challenging if not impossible to achieve” (Darwish and Roman, 2017).

Over time, the principles of nerve-sparing surgery, adopted from oncological surgery (Luka et al., 2010; Ceccaroni et al., 2010; Bellester et al., 2014), have been incorporated into the treatment of DE in order to minimise iatrogenic damage to the pelvic innervation and to reduce the risk of functional complications that could severely affect patient’s quality of life after surgery (Bellester et al., 2011; Ceccaroni et al., 2016). One of the first descriptions of a nerve sparing step by step procedure, in cases of parametrial or bowel DE, is the “Negrar method”. This method includes systematic dissection, identification and preservation of the entire pelvic neural network consisting of the hypogastric nerves, the pelvic splanchnic nerves, the inferior hypogastric plexus and the sacral roots. The milestone of this radical pelvic surgery is the fact that direct laparoscopic visualisation of the nerve fibers represents the safest way to reduce the risk of iatrogenic injuries (Ceccaroni et al., 2016). Several less invasive approaches have been described (Landi et al., 2006; Zakhari et al., 2020). Zakhari et al. (2020) proposed an interfascial approach allowing gradual separation of different anatomic layers, leaving neural fibers virtually intact beneath them.

The rationale behind these nerve-sparing techniques is that direct manipulation or stretching of nerve structures during retroperitoneal dissection can result in both mechanical disruption and late ischemic damage to nerve components. In order to reduce the possible stretching and manipulation of the nerve fibers during parametrial dissection, which may potentially lead to neurapraxia or axonotmesis, we dissected the inferior hypogastric plexus only in cases of presacral fascia infiltration.

Data emerging from our study confirms that radical hysterectomy, although nerve-sparing, is associated with a certain rate of neuro-vegetative dysfunctions, even when the procedure is pursued in compliance with the well-recognised neuro- anatomical landmarks that allow locatation of the IHP.

To the best of our knowledge, no previous study has analysed the functional outcomes after radical hysterectomy for DE using validated questionnaires. This highlights how interesting the dataset is, although limited by both a small sample size and the retrospective nature of the study. Another pitfall, despite our close selection of the sample, is represented by the lack of a standardised classification of parametrectomy for DE.

## Conclusion

The NSRH for DE, when performed with a standardised technique, is associated with an improvement of pre-operative symptoms and satisfying functional outcomes. However, despite a nerve-sparing approach, this procedure is associated with a non-negligible risk of postoperative urinary dysfunction including bladder voiding deficit, even when practiced by expert surgeons.

## References

[1] Ballester M, Dubernard G, Wafo E (2014). Evaluation of Urinary Dysfunction by Urodynamic Tests, Electromyography and Quality of Life Questionnaire before and after Surgery for Deep Infiltrating Endometriosis.. Eur J Obstet Gynecol Reprod Biol.

[2] Ballester M, Santulli P, Bazot M (2011). Preoperative evaluation of posterior deep-infiltrating endometriosis demonstrates a relationship with urinary dysfunction and parametrial involvement.. J Minim Invasive Gynecol.

[3] Brookes ST, Donovan JL, Wright M, Jackson S (2004). A Scored Form of the Bristol Female Lower Urinary Tract Symptoms Questionnaire: Data from a Randomized Controlled Trial of Surgery for Women with Stress Incontinence.. Am J Obstet Gynecol.

[4] Ceccaroni M, Clarizia R, Roviglione G (2013). Neuro-Anatomy of the Posterior Parametrium and Surgical Considerations for a Nerve-Sparing Approach in Radical Pelvic Surgery.. Surg Endosc.

[5] Ceccaroni M, Pontrelli G, Spagnol E (2010). Parametrial Dissection during Laparoscopic Nerve-Sparing Radical Hysterectomy: A New Approach Aims to Improve Patients’ Postoperative Quality of Life. Am J Obstet Gynecol.

[6] Ceccaroni M, Roberto C, Francesco B (2016). Nerve-Sparing Laparoscopic Eradication of Deep Endometriosis with Segmental Rectal and Parametrial Resection: The Negrar Method. A Single-Center, Prospective, Clinical Trial. Surg Endosc.

[7] Chiantera V, Petrillo M, Abesadze E (2018). «Laparoscopic Neuronavigation for Deep Lateral Pelvic Endometriosis: Clinical and Surgical Implications».. J Minim Invasive Gynecol.

[8] D’Avout-Fourdinier P, Lempicka M, Gilibert A (2020). Posterior rectal pouch after large full-thickness disc excision of deep endometriosis infiltrating the low/mid rectum and relationship with digestive functional outcome.. J Gynecol Obstet Hum Reprod.

[9] Darai E, Thomassin I, Barranger E (2005). Feasibility and Clinical Outcome of Laparoscopic Colorectal Resection for Endometriosis.. Am J Obstet Gynecol.

[10] Darwish B, Roman H (2017). Nerve Sparing and surgery for Deep Infiltrating Endometriosis: Pessimism of the intellect or Optimism of the Will.. Semin Reprod Med.

[11] Dindo D, Demartines N, e Clavien PA (2004). Classification of Surgical Complications: A New Proposal with Evaluation in a Cohort of 6336 Patients and Results of a Survey. Ann Surg.

[12] Ercoli A, Delmas V, Fanfani F (2005). Terminologia Anatomica versus Unofficial Descriptions and Nomenclature of the Fasciae and Ligaments of the Female Pelvis: A Dissection-Based Comparative Study.. Am J Obstet Gynecol.

[13] Filocamo MT, Serati M, Li Marzi V (2014). The Female Sexual Function Index (FSFI): Linguistic Validation of the Italian Version.. J Sex Med.

[14] Ianieri MM, Mautone D, Ceccaron M (2018). Recurrence in Deep Infiltrating Endometriosis: A Systematic Review of the Literature.. J Minim Invasive Gynecol.

[15] Knowles CH, Eccersley AJ, Scott SM (2000). Linear Discriminant Analysis of Symptoms in Patients with Chronic Constipation: Validation of a New Scoring System (KESS).. Dis Colon Rectum.

[16] Kupelian AS, Cutner A (2016). Segmental bowel resection for deep infiltrating endometriosis.. BJOG.

[17] Kyo S, Kato T, Nakayama K (2016). Current concepts and practical techniques of nerve-sparing laparoscopic radical hysterectomy.. Eur J Obstet Gynecol Reprod Biol.

[18] Landi S, Ceccaroni M, Perutelli A (2006). Hum Reprod. Laparoscopic nerve-sparing complete excision of deep endometriosis: is it feasible.

[19] Luka R, Halaska M, Robova H (2010). Nerve-Sparing and Individually Tailored Surgery for Cervical Cancer.. Lancet Oncol.

[20] Mabrouk M, Raimondo D, Arena A (2019). Parametrial Endometriosis: The Occult Condition That Makes the Hard Harder.. J Minim Invasive Gynecol.

[21] Nezhat C, Nguyen K, Ackroyd E (2020). Nerve-Sparing Modified Radical Hysterectomy for Severe Endometriosis and Complex Pelvic Pathology.. Cureus.

[22] Nieveen Van Dijkum EJ, Terwee CB, Oosterveld P (2000). Validation of the Gastrointestinal Quality of Life Index for Patients with Potentially Operable Periampullary Carcinoma.. Br J Surg.

[23] Querleu D, Cibula D, Abu-Rustum NR (2017). Update on the Querleu-Morrow Classification of Radical Hysterectomy.. Ann Surg Oncol.

[24] Raspagliesi F, Bogani G, Spinillo A (2017). Introducing nerve-sparing approach during minimally invasive radical hysterectomy for locally-advanced cervical cancer: A multi-institutional experience.. Eur J Surg Oncol.

[25] (1997). Revised American Society for Reproductive Medicine classification of endometriosis: 1996. Fertil Steril.

[26] Riiskjær M, Forman A, Kesmodel US (2018). Pelvic Pain and Quality of Life Before and After Laparoscopic Bowel Resection for Rectosigmoid Endometriosis: A Prospective, Observational Study.. Dis Colon Rectum.

[27] Uccella S, Gisone B, Serati M (2018). Functional outcomes of nerve-sparing laparoscopic eradication of deep infiltrating endometriosis: a prospective analysis using validated questionnaires.. Arch Gynecol Obstet.

[28] Zakhari A, Mabrouk M, Raimondo D (2020). Keep Your Landmarks Close and the Hypogastric Nerve Closer: An Approach to Nerve-Sparing Endometriosis Surgery.. J Minim Invasive Gynecol.

